# Probabilistic inference and Bayesian‐like estimation in animals: Empirical evidence

**DOI:** 10.1002/ece3.11495

**Published:** 2024-07-11

**Authors:** Thomas J. Valone

**Affiliations:** ^1^ Department of Biology Saint Louis University Saint Louis Missouri USA

**Keywords:** Bayesian updating, cognition, decision‐making under uncertainty, patch use, probability distribution

## Abstract

Animals often make decisions without perfect knowledge of environmental parameters like the quality of an encountered food patch or a potential mate. Theoreticians often assume animals make such decisions using a Bayesian updating process that combines prior information about the frequency distribution of resources in the environment with sample information from an encountered resource; such a process leads to decisions that maximize fitness, given the available information. I examine three aspects of empirical work that shed light on the idea that animals can make such decisions in a Bayesian‐like manner. First, many animals are sensitive to variance differences in behavioral options, one metric used to characterize frequency distributions. Second, several species use information about the relative frequency of preferred versus nonpreferred items in different populations to make probabilistic inferences about samples taken from populations in a manner that results in maximizing the likelihood of obtaining a preferred reward. Third, the predictions of Bayesian models often match the behavior of individuals in two main approaches. One approach compares behavior to models that make different assumptions about how individuals estimate the quality of an environmental parameter. The patch exploitation behavior of nine species of birds and mammals has matched the predictions of Bayesian models. The other approach compares the behavior of individuals who learn, through experience, different frequency distributions of resources in their environment. The behavior of three bird species and bumblebees exploiting food patches and fruit flies selecting mates is influenced by their experience learning different frequency distributions of food and mates, respectively, in ways consistent with Bayesian models. These studies lend support to the idea that animals may combine prior and sample information in a Bayesian‐like manner to make decisions under uncertainty, but additional work on a greater diversity of species is required to better understand the generality of this ability.

## Abstract 2

I examine empirical work that sheds light on the idea that animals can make decisions in a Bayesian‐like manner. I show evidence that animals are sensitive to variation in reward options and can make probabilistic inferences, two foundations of Bayesian‐like estimation. I also show that Bayesian models often predict the behavior of individuals in different environmental contexts.

## 1 INTRODUCTION

What surprises us, however, is that there are critics who resist our assumption that animals use probabilistic information as instantaneous clues to predict their next move … (Gowaty & Hubbell, 2013)

A straightforward assumption is that animals can make better decisions through the use of … Bayesian reasoning. This requires that the animal's cognitive system is able to calculate, represent and use probabilities and accumulate information about the different choice options. The evidence is scant and the current view is that this capacity in animals is generally rather poor (Budaev et al., 2019)

The above quotes represent the spectrum of thoughts regarding how animals make decisions under uncertainty. Animals make many decisions without perfect knowledge of their world; the quality of encountered food patches, mates, and competitors is often unknown, as is the likelihood of predator attack. Individuals, therefore, must gather information from their sensory systems in order to update estimates of various aspects of their local environment. Of this, there is little doubt (e.g., Dall et al., 2005; Schmidt et al., 2010). However, how individuals use such information to formulate environmental estimates is less clear. Note that throughout, I use the word “estimate” as a representation of information about the world that animals use in order to act in an appropriate (fitness‐enhancing) manner.

Bayesian probability theory provides an approach to estimate the value of an unknown parameter based on available information (Kilpatrick et al., 2021; Winkler, 1972). To see how this works, consider an individual searching for food hidden in a patch. Let's say the environment contains many patches that vary in the number of items they contain (i.e., their quality), and so an individual might learn the frequency distribution of different patch types. For simplicity, assume that only three types of patches exist (low, medium, or high quality) and that 50% of the patches are of low quality, 40% are medium, and 10% are high. A forager does not know the quality of an encountered patch but does know the frequency distribution of patch types in the environment, a parameter known as the prior distribution. Our forager now begins to search for food items. This process provides sample information (time spent, number of items harvested) about the quality of the patch. Bayes' theorem provides a formal way to combine sample information with a prior distribution to produce a posterior distribution that provides the likelihoods (estimates) that the patch is of a particular quality (low, medium, or high). Our forager can use this information to decide how long to stay in the patch: either continue exploiting or abandon it in search of one of higher quality. Two key elements emerge from this simple example: (i) Bayesian updating assumes the use of a prior distribution that represents the frequency distribution of types of things in the environment (here, food patches) and (ii) individuals combine sample and prior information to generate a posterior frequency distribution that provides an estimate of the unknown parameter. As such, a form of probabilistic reasoning is involved in making decisions when confronted with an unknown environmental parameter.

Much theory about how animals make decisions under uncertainty is based on formal models of Bayesian updating (McNamara et al., 2006; McNamara & Houston, 1980). Critics, however, argue that Bayesian inference is computationally demanding, requiring calculations using probability distributions, and so Bayesian decision‐making may be too difficult to employ, especially in complex environments (Budaev et al., 2019; Field & Bonsall, 2018; Lange & Dukas, 2009). Furthermore, empirical evidence of Bayesian estimation in (cognitively advanced) humans is also the subject of much debate (e.g., Bowers & Davis, 2012; Eberhardt & Danks, 2011; Griffiths & Tenenbaum, 2006; Icard, 2018; Pothos et al., 2021; Vul et al., 2014).

Given the ongoing discourse regarding the appropriateness of assuming that animals can estimate environmental parameters in ways that mimic Bayesian updating, it seems an appropriate time to examine the empirical literature on decision‐making under uncertainty in animals. My approach is to examine empirical studies that provide evidence that animals may estimate unknown parameters in ways resembling Bayesian estimation. It is not meant to be a critical review or explicit test of how animals make decisions under uncertainty. Rather, I present empirical work to address the question, “What evidence suggests animals might be able to make Bayesian‐like estimates of unknown parameters?”, illustrating the different empirical protocols employed.

Because Bayesian inference involves the use of probability distributions, I begin by briefly summarizing work showing that many animals are sensitive to probabilistic variation in reward distribution options and then turn attention to recent work on the ability of animals to make probabilistic inferences, two important building blocks of Bayesian‐like decision making under uncertainty. Finally, to more directly shed light on whether animals estimate environmental parameters in a Bayesian‐like manner, I examine several studies that compare predictions of Bayesian models to observed behavior. I searched for appropriate papers using Google Scholar and associated references therein (Date accessed Oct 1, 2023). Search terms used included “animal” with “risk sensitivity,” “probabilistic variation,” “probabilistic inference,” “statistical inference,” “Bayesian updating,” and “decision‐making under uncertainty.”

This paper extends my previous paper on Bayesian estimation in animals (Valone, 2007) by including work on Bayesian estimation published since that time as well as by reviewing studies on sensitivity to probabilistic variation and probabilistic inference in animals.

## 2 SENSITIVITY TO PROBABILISTIC VARIATION

If animals estimate the value of environmental parameters in a Bayesian‐like manner by combining prior and sample information, they must, in some manner, store and use information in probability distributions. Probability distributions summarize the probability that a parameter takes on a specific value. Continuing with our earlier example, a simple probability distribution might be that there is a 50% probability that a food patch contains two items, a 40% probability that it contains eight items, and a 10% probability that it contains 20 items (i.e., low, medium, and high quality). All such distributions can be characterized in part, by their statistical mean and variance.

There has been much work examining whether animals are sensitive to statistical variance in choice options, a phenomenon known as risk sensitivity, where “risk” denotes variance in a distribution, not predation risk. Protocols generally offer individuals two options to obtain a reward, typically food or water. Individuals can select only one option in a given experimental trial. The two options have the same mean reward but differ in their variance. Most often, one option has no variance—it represents the constant (predictable) reward, whereas the other is associated with some variance because multiple values are possible with different probabilities. Most often, there is a 50% probability of a high reward and a 50% probability of a low reward, which often is no resource. Before testing to determine whether individuals prefer one option over the other, researchers provide individuals with experience about the two options, so they have an opportunity to learn the probability distribution of each (i.e., mean and associated variance). After testing, if individuals exhibit no preference for options that have the same mean but differ in variance, they are said to be risk‐invariant. Individuals who significantly prefer the low (often no) variance option are called risk‐averse, while those who significantly prefer the option with higher variance are called risk‐prone or risk‐seeking.

Such protocols have been used in scores of mostly laboratory studies on dozens of species, and most animals exhibit significant sensitivity to risk. Kacelnik and Bateson (1996) reviewed 59 papers that examined over two dozen species of birds, bees, mammals, fishes, and a wasp. All but two bee species (*Apis mellifora*, *Bombus fervidus*) exhibited sensitivity to risk in at least one study, with a vast majority of species exhibiting risk aversion. More recent work has focused largely on primates, a group underrepresented in earlier work. Captive orangutans (*Pongo abelli*), gorillas (*Gorilla gorilla*), bonobos (*Pan paniscus*), chimpanzees (*Pan troglodytes*), mangabey monkeys (*Cercocebus torquatus*), capuchin monkeys (*Sapajus apella*), four species of macaques, and three species of lemurs are also sensitive to risk (De Petrillo et al., 2015; De Petrillo & Rosati, 2019; Hayden & Platt, 2007; Haun et al., 2011; MacLean et al., 2012; Rivière et al., 2018; Rosati & Hare, 2012; see De Petrillo & Rosati, 2021 for review). Interestingly, while most primates exhibited risk‐aversion, chimpanzees and orangutans tend to be risk‐prone more often in their behavior than the other primates studied (Haun et al., 2011; Haux et al., 2023; Rosati & Hare, 2012). While the above studies revealed interspecies variation in risk sensitivity, intraspecific variation has also been found. Work on Siberian jays (*Perisoreus infaustus*) has shown that social class can influence risk sensitivity within a species; adult breeders tended to be risk‐prone, while nonbreeders were risk‐averse (Ratikainen et al., 2010). Despite this variation in risk tolerance or preference, the important point is that a wide range of animals (now over 30 species) are sensitive to variation in reward option—an important foundation for probabilistic reasoning.

## 3 REASONING ABOUT PROBABILITIES

Can animals calculate, represent, and use probabilities when making decisions? Probabilistic inference requires that individuals make logical inferences about the probable identity of a sample from a population by incorporating information about probabilities. Such an ability to draw inferences about probabilities (also known as statistical reasoning) is important because it allows individuals to make knowledgeable predictions in the face of uncertainty.

A standard approach to assess the ability to make probabilistic inferences in animals involves presenting an individual with two populations that differ in the proportion of two food items: one preferred over the other (the nonpreferred item). The subject views the experimenter randomly drawing an item out of each population in a manner that keeps the item hidden from the subject. The subject can then select one of the drawn sample options. In order for the subject to most likely obtain a preferred rather than nonpreferred food item, it must use proportional reasoning to assess the relative frequencies of the preferred items in each population and then form an expectation about the sample drawn to assess which option has a higher probability of being a preferred food item. Note that probabilistic inference requires that an individual draw inferences about *relative* quantities rather than use a simpler heuristic, such as selecting the sample from the population with the highest *absolute* number of preferred items. In other words, individuals must use both the numerator and denominator in making their assessment of the frequency of each type of item in each population. Thus, an individual demonstrating true probabilistic inference would prefer a single sample from a population with 10 preferred and five nonpreferred items (67% preferred items) compared to a single sample from a population with 15 preferred and 20 nonpreferred items (37.5% preferred items) even though the latter contains more preferred items. Much of this empirical work has focused on mammals (mostly primates). Here, I highlight several examples.

Rakoczy et al. (2014) examined the ability of gorillas, orangutans, chimpanzees, and bonobos to make probabilistic inferences about single‐item samples from populations. These experiments varied the relative proportion of preferred and nonpreferred food items in two populations. Each population was contained in a glass jar, with the two food items being different colors but of the same relative size and shape. In the first experiment, each population had 80 items, but population A contained 80% preferred items while population B contained 20% preferred items. Individuals of all species chose the sample from population A significantly more often than that from population B, even on the first trial (and so ruling out learning over trials), demonstrating the ability to distinguish differences in relative probabilities and make a correct logical inference regarding the relative likelihood that the sample is a preferred item.

Two follow‐up experiments varied both the absolute number of preferred items and their frequency in the two populations. In the first set of trials, population A contained 20 preferred items and 0 nonpreferred items (100% preferred), whereas population B contained 100 preferred and 200 nonpreferred items (33% preferred). In the second set of trials, population A contained 12 preferred and three nonpreferred items (80% preferred), whereas population B contained 100 preferred and 400 nonpreferred items (25% preferred). In the first of these experiments, individual orangutans, chimpanzees, and bonobos (but not gorillas) significantly more often chose the sample from population A, the population with the higher relative frequency of preferred items, even though it contained a lower absolute number of preferred items while in the second experiment, all of the apes selected the sample from the population with the higher relative frequency of preferred items significantly more often than the sample from the other population. In all of the above experiments, individuals in each species exhibited a statistical preference for the sample from population A in the first trial of the experiments, and so the patterns observed cannot be explained by reinforcement learning over trials. Other experiments ruled out visual, auditory, olfactory, and tactile information about the sample. Eckert et al. (2018) conducted a similar set of experiments on chimpanzees and also found they exhibited probabilistic inference by using information about the relative frequency of preferred items in two populations.

Tecwyn et al. (2017) used similar procedures in several experiments to examine probabilistic reasoning in New World capuchin monkeys (*Sapajus* spp.) In experiment 1, the preferred:nonpreferred ratio of food items in population A was 240:60 (80% preferred) while in population B, the ratio was 60:240 (20% preferred). In experiment 2, the ratio was 32:8 (80% preferred), whereas in population B, it was 60:240 (20% preferred but contained a larger absolute quantity of preferred items). In these two experiments, individuals overall significantly preferred the sample from population A, even when it contained a fewer absolute number of preferred items (experiment 2); results again were consistent with the ability to make correct probabilistic inferences in order to most likely obtain a preferred food item.

However, in these two experiments, individuals might have used a simpler heuristic, such as avoiding the sample from the population with the larger absolute quantity of nonpreferred items. To rule out this alternative, the research team conducted one final experiment in which the ratio was 100:200 (33% preferred) in population A, while in population B, it was 22:200 (10% preferred). In this experiment, individuals selected the sample from population A more often than B, but the difference was only marginally significant (*p* < .07). So the researchers cannot conclusively rule out the use of a simpler heuristic based on avoiding the absolute number of nonpreferred items. Despite this, these experiments overall provide initial support for the hypothesis that these monkeys, like great apes, can also make logical inferences about probabilities but perhaps not at the same level as great apes. Also, notably, there was no increase in the selection of the sample from population A over trials in the above experiments, indicating that individuals did not learn to make better choices over trials through reinforcement.

While most work on probabilistic inference in mammals has been conducted on primates, Caicoya et al. (2023) examined captive giraffes (*Giraffa camelopardalis*). Like most of the studies above, they visually presented individuals with two populations that had different relative frequencies of preferred and nonpreferred food items over a series of tests. In test 1, the preferred:nonpreferred ratio of food items in population A was 100:20 (83% preferred), whereas in population B, it was 20:100 (16% preferred). In test 2, the ratio was 20:4 (83% preferred) in population A and 20:100 (16% preferred) in population B. In test 3, the ratio was 57:63 in population A (48% preferred) and 3:63 in population B (4.5% preferred). All individuals in all three experiments performed above chance and significantly preferred the sample from the population that had a higher relative frequency of preferred items. Such work extends evidence of probabilistic inference in mammals outside primates and, unlike primates, to a mammal with small relative brain size, suggesting that probabilistic inference in mammals may not require a relatively large brain.

Outside of mammals, work on probabilistic inference has focused on birds. The strongest evidence of probabilistic inference comes from work on kea (*Nestor notabilis*), a parrot native to New Zealand. Bastos and Taylor (2020) examined six males in which individuals observed two populations that differed in the proportion of two items, in this case, different colored tokens. One token, the preferred item, was black and could be traded for food (rewarding item). The other token, the nonpreferred item, was orange and could not be traded for food (nonrewarding).

Individuals observed two populations of tokens in different glass jars and so could estimate the relative proportion of token types in each. Subjects viewed an experimenter removed one token (not visible to the subject) from each jar and offered a choice of the two samples. In three different experimental conditions, the populations differed in the number and relative frequency of tokens across a series of experiments. In the first condition, population A contained 100 preferred items and 20 nonpreferred items (83% rewarding), whereas population B contained 20 preferred and 100 nonpreferred items (17% rewarding). In the second condition, population A contained 20 preferred and four nonpreferred items (83% rewarding), whereas population B contained 20 preferred and 100 nonpreferred items (17% rewarding). In condition 3, population A contained 57 preferred and 63 nonpreferred items (48% rewarding), whereas population B contained three preferred and 63 nonpreferred (4.5% rewarding).

Four of six subjects performed above chance in selecting the sample from population A in conditions 2 and 3, whereas three did so in condition 1, demonstrating that individual kea appears to be able to use the relative frequency of items to obtain rewards (make the best choice of two options to obtain a reward) and not a simpler quantity heuristic such as selecting the sample from the population with a greater absolute number of rewarding tokens or avoiding the sample from the population with a greater absolute number of unrewarding tokens. Analysis of all data from first trials over all experiments displayed similar patterns and so helps to rule out the alternative explanation of associative learning over trials in the experiments.

The above studies indicate that many animals are sensitive to variance in reward options of different choices and that some mammals and birds appear able to make probabilistic inferences. Such abilities do not provide evidence of Bayesian‐like estimation but rather provide insight into the logical capabilities animals can possess when making decisions under uncertainty. In the next section, I review how researchers have more directly investigated whether animals use Bayesian‐like processes to estimate the quality of encountered resources.

## 4 BAYESIAN‐LIKE ESTIMATION IN ANIMALS

Two common approaches have been used to investigate the information individuals use to make decisions under uncertainty. The first relies on the common scientific approach of creating alternative hypotheses, in this case models of information use, involving how individuals might make decisions under uncertainty: the models generate different predictions that are compared to data (Platt, 1964). The second approach involves the manipulation of prior distribution information by allowing different individuals the opportunity to learn different frequency distribution of resources. When such manipulations significantly affect behavior in predicted ways, it suggests that information about prior distributions is influencing decision‐making under uncertainty. I review select examples of both approaches to illustrate their use in providing support for Bayesian‐like updating in animals.

## 5 COMPARING MODEL PREDICTIONS TO BEHAVIOR

Much empirical work on Bayesian‐like estimation has examined foragers exploiting food patches. In this approach, discrete resources are hidden within a food patch so that a forager does not know patch quality (the total number of items it contains) and experiences diminishing returns as food is harvested. Valone and Brown (1989) describe how patterns of resource exploitation from paired patches that only differ in initial resource amount can provide insight into the exploitation strategy and information used by the forager who exploits them, as follows. When an individual encounters two patches, in close proximity, that are identical except for the initial resource amount, an ideally informed omniscient forager should exploit each patch down to the same quitting harvest rate to maximize the energy intake rate (Charnov, 1976). When harvest rate within a patch is a function of resource density only, and foragers use random search within a patch, then equal quitting harvest rates (the harvest rate just at patch departure) correspond to equal giving up densities (GUD) of food in each patch (Brown, 1988). Note that in order to equalize GUD's, the forager must spend more time and remove more food items from the richer patch (the patch with more food items) than the poorer patch. As such, biasing search effort toward the richer patch results in positive density‐dependent exploitation in which the ratio of the GUD's (rich patch divided by poor patch) is smaller after exploitation (i.e., the densities become more similar) than the ratio of the initial densities in these patches.

If a forager does not, or cannot, distinguish differences in the density of food in the paired patches, it should treat the patches the same, use a fixed‐time strategy, and spend the same amount of search time in each patch (McNair, 1983). This behavior represents a type of ‘null model’ because it implies no ability or attempt to bias effort toward the richer patch. This is how an uninformed forager would behave. Such a “fixed time” foraging strategy will result in density‐independent exploitation because the ratio of GUD's in the two patches will not become more equal after exploitation (they will remain equal to the ratio of the initial food abundances).

Most patch exploitation by individuals likely lies between these two information extremes (perfect knowledge of patch qualities and no use of information to bias effort toward exploiting the richer patch during exploitation). Valone and Brown (1989) describe two (of potentially many) possible foraging strategies for such individuals. One possibility is that individuals use only sample information obtained from an encountered patch to estimate its quality and so do not use prior information. By using all the sample information obtained in a patch, the “assessor” forager bases its patch leaving the decision on the average harvest rate from the patch. This rate will initially be high for patches rich in resources and much lower for patches depauperate of resources (low‐quality patches). When both a rich and poor patch is exploited down to the same resource density, the average harvest rate of the two patches will not be equal. So the individual will need to spend additional time and consume additional resources from the rich patch in order to reduce its average harvest rate in the patch to the same quitting harvest rate level as the poor patch. As such, the forager will exhibit positive density‐dependent foraging, biasing effort toward the richer patch, but the giving up the density of the rich patch will be left below the giving up the density of the poor patch.

Finally, a Bayesian forager combines sample information with knowledge of a prior distribution of resources (patch types) in the environment and will bias search effort toward the richer patch but will leave the GUD of the richer patch higher than the GUD of the poorer patch (Kilpatrick et al., 2021; Valone & Brown, 1989). Why? The Bayesian estimator used to decide when to abandon a patch is assumed to be the mean of the posterior distribution. By definition, low‐quality patches contain fewer resources than the mean patch in the environment, while rich patches contain more and so when a forager enters a low‐quality patch, the Bayesian estimator at the start of exploitation (the mean of the prior distribution) initially lies above the actual patch quality. In other words, upon patch entry, the forager estimates the patch to be of higher quality than it actually is because it does not know it is a poor‐quality patch. As sample information is collected, the forager learns that the patch is of lower quality than its initial estimate, but the estimator (the mean of the posterior distribution) remains at a value higher than the actual patch density even at patch departure. In contrast, when a forager enters a rich patch, the Bayesian estimator initially lies below the actual patch quality (because the forager does not know it is a rich patch). During exploitation, the estimator tends to decline as resources are harvested, but the estimator (mean of the posterior distribution) will remain below the actual patch quality, even at patch departure. That is, the forager will always estimate a rich patch to have a lower density of food than it actually does due to the use of the prior distribution information. Thus, poor patches will have lower GUD's than rich patches: rich patches will be underutilized relative to poor (for details, see Valone & Brown, 1989; Kilpatrick et al., 2021).

These four foraging and information use strategies make unique sets of predictions about the exploitation patterns of paired food patches (Table 1) and have formed the basis for many empirical tests of information use by foragers that differ in initial food abundance (quality). These studies present foragers with paired patches and examine exploitation patterns by collecting GUDs (Valone, 2006). The patch use patterns of four birds in four different orders (red knot [*Calidris canutus*]; O. Charadriiformes, Inca dove [*Columbina inca*]; O. Columbiformes, common crane [*Grus grus*]; O. Gruiformes; and lesser spotted woodpecker [*Dendrocopos minor*]; O. Piciformes) (Alonso et al., 1995; Olsson et al., 1999; Valone, 1991; van Gils et al., 2003), and five mammals in two orders (Merriam's kangaroo rat [*Dipodomys merriami*], Arizona pocket mouse [*Perognathus amplus*], round‐tail ground squirrel [*Xerospermophilus tereticaudus*]), and degus ([*Octodon degu*]; all O. Rodentia, and chimpanzees; O. Primates) (O'Bryan et al., 2020; Valone & Brown, 1989; Vásquez et al., 2006) are consistent with Bayesian foraging: they bias exploitation effort toward the richer patch but underutilized rich relative to poor patches (Table 1).

**TABLE 1:** Predicted patterns of patch use for paired patches that differ in initial resource amount for four different information use foraging strategies.

	Positive density dependent resource harvest	GUD in rich patch relative to poor
**Foraging strategy**	Resource harvest?	Relative to poor
Omniscient	Yes	Equal GUDs
Fixed time	No	Higher GUD in rich
Assessor	Yes	Lower GUD in rich
Bayesian	Yes	Higher GUD in rich

Here, I highlight one such study in detail, the work of Vásquez et al. (2006) on captive degus, both to illustrate the approach and because the findings illuminate how individuals can alter their foraging strategy as they gain information from their environment. Most studies of paired patch exploitation randomly assign a different initial food density to each of the paired patches on each trial so that foragers do not have information about patch quality prior to sampling. Vásquez et al. (2006), however, fixed resource renewal amounts in a specific patch over repeated trials. Thus, individuals could learn, over the course of trials, the location of the rich and poor patch and then use that information to exploit each patch. This experimental protocol allows the opportunity to observe changes in patch exploitation and information use over time.

In particular, wild‐caught individuals were brought into the laboratory and offered pairs of food patches that differed in initial food amount mixed into a fixed amount of sand. Individuals could exploit the patches for 4–5 h each day, after which the GUDs were collected. At the start of each day, each patch was replenished with the same amount of food so that the location of the rich patch (12 g of food) and poor patches (8 g of food) was fixed each day, providing the opportunity for each forager to learn this aspect of the environment with experience. Each of the 24 individuals was tested for 21 days.

Overall, degus appeared to use a fixed time foraging strategy on days 1–4 because they did not bias search effort toward the richer patch. On days 5–16, individuals biased search effort toward the richer patch because they exhibited positive density‐dependent foraging but were still underutilizing the rich patch relative to the poor patch, a pattern characteristic of Bayesian foraging. Then, on days 17–21, individuals equalized GUDs of the two patches and so exhibited an omniscient foraging pattern. Thus, over 21 days, individuals exhibited three different patch‐use strategies as they learned more about the conditions and renewal of resources in the experimental patches.

This experiment shows flexibility in food patch exploitation strategy based on different information use that makes sense: on days 1–4 individuals had no experience with the experimental design and so needed experience to learn the difference in patch qualities. By day 5, however, individuals were consistently biasing foraging efforts toward the richer patch after they had the opportunity to learn about the frequency distribution of patch types in the environment but were still unable to fully equalize GUDs. By day 17, individuals apparently had acquired sufficient experience with the initial food abundances to behave as fully informed prescient foragers by fully equalizing GUDs of the rich and poor patches.

In sum, nine species of mammals and birds across six orders have exhibited patterns of patch use consistent with predictions of Bayesian estimation and form the greatest evidence in support of a Bayesian‐like estimation process in a diverse set of animals. Note, however, that the GUD approach using paired patches makes several assumptions (Bedoya‐Perez et al., 2013), including that foragers can keep track of the quality of each patch during exploitation. Next, I review an alternative and perhaps the strongest approach to investigating Bayesian estimation in animals: the manipulation of prior information.

## 6 MANIPULATING PRIOR DISTRIBUTIONS

A central feature of Bayesian estimation is the use of a prior distribution as a source of information to aid in estimating an unknown parameter. Thus, one can predict that if individuals learn (and use) different prior distributions, they will often behave differently, in predictable ways, even if they experience the same sample information.

Lima's (1984) examination of the feeding behavior of free‐living downy woodpeckers (*Picoides pubescens*) on artificial patches exemplifies how individuals exposed to different prior distributions are predicted to behave differently if using such information and so can provide insight into their information use. In brief, individuals were trained over weeks, experiencing dozens of patches that contained covered holes that might contain a food item on one of three different prior distributions of rewards in patches; in each case, 50% of the patches contained no food (all covered holes with no food) while the other 50% were either completely full (24 of 24 covered holes with food), ½ full (12 of 24 randomly selected covered holes with food), or ¼ full (six of 24 randomly selected covered holes with food). Lima focused on the sampling behavior of individuals on completely empty patches in each environment. If individuals learned and used information about the prior distribution (frequency distribution of patch types), they should sample empty patches to a different degree in the different environments before deciding to abandon them, and that is exactly what the birds did. Using knowledge of the prior distributions, individuals could most efficiently exploit completely empty patches by sampling 1, 3, and 6 empty holes, respectively, in the completely full, ½ full, and ¼ full, environments, respectively, before departing it. In fact, on average, the birds sampled about 1, 4, and 6 empty holes, respectively, in these three environments before abandoning an empty patch, compelling evidence that their behavior had been influenced by the distribution of food in the patches in each environment in a predictable way. A similar experiment in the laboratory using starlings (*Sturnus vulgaris*) sampling empty and differentially filled patches found qualitatively similar results (Lima, 1985). Both studies indicate that knowledge about the distribution of food among patches in the environment influenced sampling behavior of these birds in a manner consistent with Bayesian‐like estimation.

Valone (1992) studied the behavior of free‐living, black‐chinned hummingbirds (*Archilochus alexandri*) exploiting food patches in which rewards were hidden in patches of 12 artificial flowers. Each bird experienced one of two environments over 1–3 weeks, experiencing dozens of patches, which represented different prior distributions of patch types. The simple environment contained two patch types in equal frequency (two or five provisioned flowers), whereas the complex environment contained eight equally frequent patch types (zero to seven provisioned flowers). The mean patch was identical in the environments, 3.5 of 12 provisioned flowers. Similar to Lima's work, the behavior of birds prior to patch departure was characterized and again focused on each individual's tolerance of unrewarded search (number of empty flowers probed before patch departure).

Two predictions were examined, based on whether individuals only used sample information from a patch to estimate its quality or instead combined sample information with knowledge of the frequency distribution of patch types, that is, the prior distribution. If birds were only using sample information to estimate patch quality, their patch estimates should increase with the number of rewards found, and so such individuals should be more tolerant of unrewarded search prior to patch departure. Therefore, one might expect a positive correlation between the number of rewards found in a patch and the number of unrewarded flowers searched just prior to departure from it. However, if individuals have learned the prior distributions, they should be less and less tolerant of unrewarded search as they obtain more rewards because they know a patch contains a maximum of either 5 or 8 provisioned flowers, depending on the environment experienced. Such use of prior information allows one to predict there will be a negative relationship between number of rewards found and tolerance of unrewarded search.

Six of the seven birds trained in the simple environment exhibited a negative correlation between number of rewards found and tolerance of unrewarded search in a patch after experiencing about 25 patches; four of these correlations were significant. In the complex environment, behavior was more variable. Two birds exhibited a significant negative correlation between rewards found and tolerance of unrewarded search, again after experiencing about 25 patches, while three others exhibited a significant positive correlation. Overall, these data are consistent with the notion that many of the birds appeared to use information about the frequency distribution of rewards in the environment while exploiting the artificial patches, especially in an environment that was simpler to learn, as one might expect.

Biernaskie et al. (2009) studied bumblebees exploiting patches of 12 artificial flowers in the laboratory. They also trained individuals in one of two environments by exposing them to at least 45 patches in about 2–3 h. In the “uniform” environment, all patches had five of 12 randomly provisioned flowers, whereas in the high variance environment, half the patches contained one randomly placed reward while half contained nine randomly placed rewards. Note that again, in both environments, the mean patch was identical (five provisioned flowers). Like Valone's work, the research team measured the average tendency of an individual to remain on a patch during exploitation as a function of the number of rewards it had found. If individuals were using prior information about the frequency distribution of patch types, individual tendency to remain on a patch should decline with the number of rewards found in the uniform environment because each time a reward was consumed, the individuals could know that one less reward remained in the patch. And, when it had consumed the fifth reward, it should immediately depart the patch. However, in the high variance environment, individuals who have found a second reward, can know that the current patch contains an additional seven rewards. So, its propensity to remain in the patch should increase when it finds that second reward and decline thereafter as it exploits the remaining rewards in the patch, leaving after it finds the 9th reward.

Biernaskie et al. (2009) found significant differences in the behavior of individuals trained in the two environments. Individuals in the uniform environment were less and less likely to remain on a patch as they found more rewards. However, individuals in the high variance environment were least likely to depart the patch after finding the second reward, as predicted if they were using information about the frequency distribution of patch types in the environment.

These studies illustrate that both vertebrates and invertebrates behave in ways that are consistent with Bayesian‐like information use about the distribution of food among patches in the environment when exploiting artificial food patches. Of course, because neither study examined other forms of information use, alternative explanations for the observed behaviors cannot be ruled out.

Besides feeding, there are other circumstances when individuals make decisions under uncertainty, such as reproduction. For many species, individuals must select a mate, and there can be much variation in mate quality (potential reproductive output, genetic quality) so fitness can be affected by the decision. When individuals encounter potential mates one at a time, for each one, they must decide whether to mate with that individual or reject them and continue searching for a higher‐quality mate. Gowaty and Hubbell (2009) formalized how individuals can make these decisions in their “switch point theorem” model. The model assumes individuals can instantaneously and accurately assess the quality of an encountered mate and also possess knowledge of the frequency distribution of prospective mate quality types in the population, which it uses in deciding whether to accept or reject an encountered potential mate. If the population is dominated by high‐quality mates, the focal subject can maximize its fitness by being highly selective, and so, commonly reject low‐quality individuals. Alternatively, if the population is dominated by low‐quality individuals, the focal individual should be less selective, widen its acceptance threshold, and so, commonly mate with a low‐quality individual. As such, differences in the frequency distribution of mates can explain differences in mate choice preferences.

Balaban‐Feld and Valone (2017) tested the switch‐point theorem model by raising individual males in populations that contained different frequency distributions of female qualities. Males were raised in population environments where they could see, but not mate with, high‐ and low‐quality females in different frequencies, either 75%, 50%, or 25% high quality. Each male was then tested by giving him the choice of one high‐ and one low‐quality female to court.

The courtship preference of males was affected by their rearing environment in ways consistent with the switch‐point theorem model: males who had been raised in the 75% high‐quality female population were most selective, strongly biasing courtship toward the large females. Males raised in the 25% high‐quality female population were the least selective in biasing their courtship preference toward the high‐quality female. As such, these results indicate that mate courtship behavior was influenced by the frequency distribution of female qualities that individuals experienced prior to courtship and so are qualitatively consistent with the predictions of a Bayesian model.

## 7 DISCUSSION

Bayesian probability theory provides a logically consistent method for probabilistic inference under uncertainty (Kilpatrick et al., 2021; McNamara & Houston, 1980). Bayesian estimation combines prior and current information in an updated conditional (posterior) probability distribution and so contains all available information about an environmental parameter; it has been shown to provide estimates that maximize fitness, and so it makes sense that it is the approach used by theoreticians to model decision‐making under uncertainty (McNamara & Houston, 1980). Selection should favor individuals who best combine past with current knowledge when making decisions under uncertainty and so would favor individuals who behave in a manner like a Bayesian theoretician (McNamara et al., 2006).

Bayesian‐like inference by animals, however, requires the ability to cognitively process information about the environment in probability distributions. One way probability distributions are characterized is by their variance, and abundant empirical work indicates that many animals are sensitive to variance in reward options, often preferring to avoid the option with associated variance. Such data supports the conclusion that many animals are capable of distinguishing variance in options and so are sensitive to differences in one metric used to characterize probability distributions. Furthermore, several species of mammals and birds appear to be capable of probabilistic inference; they can distinguish differences in the relative probability of samples drawn from different populations and often correctly select the option that yields the higher probability of obtaining a preferred reward. Such abilities demonstrate cognitive abilities consistent with an ability to reason about probabilities in ways consistent with Bayesian‐like reasoning.

Empirical work more directly examining Bayesian‐like updating in animals has involved two approaches: comparing predictions of different models of information used to behavior and manipulating priors. The first approach has been more widely adopted. Nine species of solitary foraging mammals and birds exhibit patch use patterns consistent with the predictions from Bayesian models of patch estimation. Other work, not explicitly examined here, has also compared Bayesian model predictions to the behavior of individuals selecting between two spatial locations of unknown quality (Arganda et al., 2012; Pérez‐Escudero & de Polavieja, 2011) and antipredator behavior of prey (Sutton et al., 2021; Sutton & O'Dwyer, 2018). The choice behavior of several species of fishes and ants as well as predator escape behavior of several species of birds and white‐tailed deer (*Odocoileus virginianus*) are well described by such Bayesian models (Arganda et al., 2012; Pérez‐Escudero & de Polavieja, 2011; Sutton et al., 2021; Sutton & O'Dwyer, 2018).

The second approach examining Bayesian‐like estimation manipulates the experience of individuals so that they learn different distributions of resources (prior distributions) and then compare their behavior. Such work on bees and several species of birds indicates that individuals learning different distributions do behave differently, in predictable ways, even after obtaining similar sampling information, which often involves unrewarded search in a food patch. Such studies provide strong support for the idea that animals learn about the frequency distribution of rewards in their environment and combine that information with sample information when making decisions under uncertainty.

I believe three factors likely contribute to the doubts expressed in the literature about the ability of animals to estimate unknown environmental parameters in a Bayesian‐like manner. One factor involves work on adult humans. Adults often perform relatively poorly when asked to estimate a parameter in which they can combine prior with sample information (e.g., Cassey et al. 2016; Colombo et al., 2021; Crupi & Calzavarini, 2023; Eberhardt & Danks, 2011). If adult humans are poor Bayesian estimators, it seems reasonable to conclude that other animals cannot do well either. However, human subjects, including young children, can make logical probabilistic inferences by learning frequency distributions through repeated experience (Cosmides & Tooby, 1996; Denison & Xu, 2019; Hogarth & Soyer, 2011; Reani et al., 2018; Redher & Waldmann, 2017; Schulze & Hertwig, 2021). Thus, testing protocols may help to explain the relatively poor statistical inference performance of adults in the past when prior information was presented as text or images. Future work investigating Bayesian‐like updating should carefully consider how animals are presented with information about the frequency distribution of resources—learning frequency distributions through experience rather than from static visual information is likely more in line with how animals learn about environments.

The second factor likely creating doubt about the ability of animals to estimate environmental parameters via a Bayesian‐like process might involve variation in the cognitive abilities of animals as they relate to brain size. While cognitive ability has been measured in many ways, one common pattern across species is that there is often a positive correlation between cognitive performance and relative brain size (brain mass or volume divided by body mass; Benson‐Amram et al., 2016; Sol et al., 2005). Because Bayesian‐like estimation is assumed to require enhanced cognitive ability, one may conclude that few animals, only those with relatively large brains, may have the cognitive architecture to store and use probabilistic information to make decisions. Throughout, we have seen that much work on animal decision‐making under uncertainty has focused on species with large relative brain sizes, particularly great apes, monkeys, and parrots. However, recent work on giraffes, a species with a relatively small brain, revealed the capacity for statistical inference, and studies comparing behavior to Bayesian models have shown that a variety of relatively small‐brained taxa (e.g., woodpeckers, doves, rodents; Burger et al., 2019; Ksepka et al., 2020) behave in ways consistent with predictions of Bayesian‐like estimation. As such, relatively large brains may not be required for Bayesian‐like estimation in animals.

Finally, the third factor involved in creating doubt about Bayesian‐like decision‐making under uncertainty may be ignorance of empirical work on the subject. Perhaps, this paper will help to begin to fill in that knowledge gap.

Going forward, additional empirical work on a greater diversity of animals is required to better understand how animals make decisions under uncertainty and my hope is that this review will helps foster such work. Such a broad approach will provide greater insights into the evolution of information use and decision‐making under uncertainty. In addition, empirical work should more fully attempt to test alternative models of information use. Ideally, such work would compare predictions from Bayesian models to those from different ways animals might estimate unknown parameters, such as linear operators or other types of models (Bush & Mosteller, 1951; Kacelnik et al., 1987; Marshall et al. 2013; Rescorla & Wagner, 1972; Trimmer et al., 2011, 2012).

Future theoretical work should continue to develop alternative models of parameter estimation. For example, Higginson et al. (2018) developed a model of estimation of the current conditions of the environment based on an individual's physiological energy reserves. In brief, the model is based on avoiding death via starvation or predation (i.e., the “small bird in winter” scenario (Houston & McNamara, 1993) and requires individuals to decide how intensively to forage in each time step). Predation risk mortality increases with both greater foraging effort and energy reserves of an individual, and the environment fluctuates between two states (high or low food availability). The analysis shows that individuals who base their foraging decisions on their energy (fat) reserves can realize overwinter survival almost as high as those using Bayesian updating to estimate the state of the environment unless environmental conditions change rather quickly. Models such as these require empirical testing but may provide an instructive alternative to Bayesian models of environmental estimation.

While we may never know for sure exactly how individuals combine past and current information to make decisions under uncertainty, available evidence suggests that they do use both sources of information in a variety of contexts. A deeper understanding of how they do this will yield a better understanding of the behavior of animals that live in uncertain worlds.

## AUTHOR CONTRIBUTIONS

**Thomas J. Valone:** Conceptualization (lead); investigation (lead); methodology (lead); project administration (lead); writing – original draft (lead); writing – review and editing (lead).

## CONFLICT OF INTEREST STATEMENT

The author declares no conflicts of interest.

## Data Availability

Data sharing is not applicable to this article as no new data were created or analyzed in this study.
